# Systolic Blood Pressure and Effects of Screening for Atrial Fibrillation With Long-Term Continuous Monitoring (a LOOP Substudy)

**DOI:** 10.1161/HYPERTENSIONAHA.122.19333

**Published:** 2022-07-08

**Authors:** Lucas Yixi Xing, Søren Zöga Diederichsen, Søren Højberg, Derk W. Krieger, Claus Graff, Morten Salling Olesen, Axel Brandes, Lars Køber, Ketil Jørgen Haugan, Jesper Hastrup Svendsen

**Affiliations:** Department of Cardiology, Copenhagen University Hospital – Rigshospitalet, Copenhagen, Denmark (L.Y.X., S.Z.D., M.S.O., L.K., J.H.S.).; Department of Cardiology, Bispebjerg Hospital, Copenhagen University Hospital, Denmark (S.Z.D., S.H.).; Department of Neurology, Mediclinic City Hospital, Dubai,United Arabic Emirates (D.W.K.).; Department of Neuroscience, Mohammed Bin Rashid University of Medicine and Health Science, Dubai, United Arabic Emirates (D.W.K.).; Department of Health Science and Technology, Aalborg University, Denmark (C.G.).; Department of Biomedical Sciences (M.S.O.), Faculty of Health and Medical Sciences, University of Copenhagen, Denmark.; Department of Clinical Medicine (L.K., J.H.S.), Faculty of Health and Medical Sciences, University of Copenhagen, Denmark.; Department of Clinical Research, Faculty of Health Sciences, University of Southern Denmark (A.B.).; Department of Cardiology, Odense University Hospital, Denmark (A.B.).; Department of Cardiology, Zealand University Hospital Roskilde, Denmark (K.J.H.).

**Keywords:** atrial fibrillation, blood pressure, hypertension, mass screening, stroke

## Abstract

**Background:**

Hypertension is a well-known risk factor for atrial fibrillation (AF) and stoke, but data on the interaction between systolic blood pressure (SBP) and effects of AF screening are lacking.

**Methods:**

The LOOP Study randomized AF-naïve individuals aged 70 to 90 years with additional stroke risk factors to either screening with implantable loop recorder (ILR) and anticoagulation initiation upon detection of AF episodes ≥6 minutes, or usual care. In total, 5997 participants with available baseline SBP measurements were included in this substudy. Outcomes were analyzed according to the time-to-first-event principle using cause-specific Cox models.

**Results:**

The hazard ratio of stroke or systemic arterial embolism for ILR versus control decreased with increasing SBP. ILR screening yielded a 44% risk reduction of stroke or systemic arterial embolism among participants with SBP ≥150 mm Hg (adjusted hazard ratio, 0.56 [0.37–0.83]). Within the ILR group, SBP≥150 mm Hg was associated with a higher incidence of AF episodes ≥24 hours than lower SBP (adjusted hazard ratio, 1.70 [1.08–2.69]) but not with the overall occurrence of AF (adjusted *P*>0.05).

**Conclusions:**

The impact of AF screening on thromboembolic events increased with increasing blood pressure. SBP≥150 mm Hg was associated with a >1.5-fold increased risk of AF episodes ≥24 hours, along with an almost 50% risk reduction of stroke or systemic arterial embolism by ILR screening compared to lower blood pressure. These findings should be considered hypothesis-generating and warrant further study.

**Registration:**

URL: https://www.clinicaltrials.gov; Unique Identifier: NCT02036450.

**Novelty and Relevance**

**What Is New?**

- We show in an elderly, at-risk population that the benefits of continuous atrial fibrillation screening on stroke prevention increases with elevating systolic blood pressure.

**What Is Relevant?**

- Atrial fibrillation screening with a cutoff of systolic blood pressure ≥150 mmHg is associated with ≈ 50% stroke risk reduction, compared with usual care.

**Clinical/Pathophysiological Implications?**

High systolic blood pressure and dysregulated hypertension may assist in identifying the appropriate population who will benefit from atrial fibrillation screening.

Atrial fibrillation (AF) confers a 5-fold increased stroke risk,^1,2^ which can be effectively reduced by guideline-directed oral anticoagulation therapy.^3–5^ However, the ASSERT (Asymptomatic AF and Stroke Evaluation in Pacemaker Patients and the AF Reduction Atrial Pacing Trial) revealed in patients with cardiovascular implantable electronic device that the majority of AF episodes were asymptomatic and even the brief ones were associated with increased stroke risk.^6^ Although individuals with undiagnosed subclinical AF potentially face a greater thromboembolic risk, evidence on health benefits of AF screening and subsequent stroke prophylaxis is sparse.

Recently, the randomized trial AF detected by Continuous ECG Monitoring using Implantable Loop Recorder to prevent Stroke in High-risk Individuals (LOOP Study) found no significant stroke reduction, when elderly, at-risk individuals were screened for AF with implantable loop recorder (ILR) and offered oral anticoagulation upon AF detection.^7^ Nonetheless, the ILR screening effects seemed to differ across levels of systolic blood pressure (SBP), with a significant benefit obtained among participants in the highest SBP tertile (≥157 mm Hg). Indeed, hypertension is a well-known risk factor for AF and stroke,^8–16^ but data on the impact of SBP on AF screening efficacy are lacking.

The present study aimed to provide insights into the relation between SBP and AF screening effects, which would inform further adjustments of screening strategies.

## Methods

The study data underlying this article cannot be shared publicly for ethical reasons, but the methodology will be shared upon reasonable request to the corresponding author (J.H.S), who has full access to all the data and takes responsibility for its integrity and the data analysis.

### The LOOP Study

The LOOP Study was an unblinded, randomized, controlled trial addressing long-term continuous AF screening in elderly individuals. A detailed description of the trial design has been published previously.^17^ In brief, individuals aged 70 to 90 years with no known AF, but with a history of arterial hypertension, diabetes, previous stroke, and/or heart failure, were randomized to either ILR monitoring or usual care (the control group). At the baseline visit, blood pressure measurement was conducted at least 3 times and the mean of the last 2 was registered. During follow-up, blood pressure was taken on in-person visits scheduled annually in the first 3 years for the ILR group and at 3-year follow-up for the control group. Upon detection of elevated blood pressure, participants were advised to contact their general practitioners for further diagnosis and treatment. In the ILR group, oral anticoagulation treatment was offered when new-onset ILR-detected AF episodes lasting ≥6 minutes were detected.

The LOOP Study was registered at www.Clinical-Trials.gov (NCT02036450) and was approved by the Regional Scientific Ethics Committee for the Capital Region of Denmark (H-4-2013-025) and the Danish Data Protection Agency (30-0955). The trial was conducted in accordance with the Declaration of Helsinki. Oral and written informed consent was obtained from all participants.

The present analysis included the LOOP participants with available SBP measurements at baseline.

### Outcomes

The primary outcome was a composite end point of stroke or systemic arterial embolism. Secondary outcomes included (1) the composite of ischemic stroke, transient ischemic attack, or systemic arterial embolism and (2) all-cause death. Other outcomes of interest were AF diagnosis and ILR-detected AF episodes ≥24 hours as a post hoc analysis of the ASSERT indicated that the bulk of strokes occurred in individuals with subclinical AF duration exceeding 24 hours.^18^

Strokes, systemic arterial embolisms, and deaths were independently adjudicated by a clinical end point committee blinded to randomization assignment, as previously described.^17^ Strokes were categorized as hemorrhagic or ischemic based on brain imaging. AF diagnoses in the control group were extracted from medical records and included any detection of AF episodes during the study period and confirmed by 12-lead ECG diagnosis of AF in the ILR group was defined as any new-onset ILR-detected AF episode ≥6 minutes independently evaluated by at least 2 senior cardiologists, or any documented AF on ECG or Holter after ILR went out of service. If the AF diagnosis was established upon detection of shorter episodes, subsequent ILR-detected AF ≥24 hours was adjudicated by at least one experienced physician.

### Statistical Analysis

Baseline characteristics are presented as means with SDs for continuous variables and as frequencies and percentages for categorical variables. Distributions were compared using Kruskal-Wallis test for continuous variables and χ^2^ test for categorical variables.

Outcomes were analyzed with the time-to-first-event method and compared between groups by use of cause-specific Cox proportional-hazards model accounting for death as competing cause for all outcomes except all-cause death. The Cox model was further subjected to multivariate analysis adjusting for sex, age, body mass index, alcohol consumption, smoking pack years, concomitant medications (beta-blockers, calcium channel blockers, renin-angiotensin inhibitors, statins, diuretics, platelet inhibitors, insulins, and other antidiabetic drugs) and medical history (hypertension, diabetes, heart failure, chronic ischemic heart disease, valvular heart disease, peripheral artery disease, and previous stroke). The proportional-hazards assumption was tested with the scaled Schoenfeld residuals, and no violation was detected. Cumulative incidences were plotted for all outcomes, using the Kaplan-Meier method for all-cause death and the Aalen-Johansen method for other outcomes considering death as competing cause. Event rates (events per 100 person-years) were estimated from Poisson regression.

The interaction between SBP and the screening effect on the primary outcome was assessed by a sliding window approach iterating through increasing levels of SBP. Here, sequentially overlapping subpopulations with the size of 1500 participants each were created with a step size of 100 for each new subpopulation. The screening effect for ILR versus control, as indicated by hazard ratio (HR), was determined in each subpopulation and plotted with the *x*-axis coordinate equal to the median SBP value within that subpopulation. A nonparametric, polynomial, moving-regression plot with locally estimated smoothing was then constructed to explore the relation between SBP and the HR for the primary outcome, with exact 95% CIs. Based on the nonparametric plot, a cutoff SBP value was selected to divide the participants into 2 risk strata for subsequent outcome analyses.

The risks of primary and secondary outcomes were compared between the randomization groups within each of the 2 risk strata. For a better understanding of the primary outcome, the occurrence of hemorrhagic stroke and ischemic thromboembolism (consisting of ischemic stroke and systemic arterial embolism) within the primary outcome was further examined separately. For other outcomes of interest, groupwise comparisons were performed between the SBP risk strata. For the end point of AF diagnosis, its dependency on blood pressure was additionally assessed with penalized spline models examining SBP as a continuous variable, where the relative risk was determined with 130 mm Hg as reference. As supplementary analysis, SBP changes for 3-year follow-up versus baseline were calculated and compared between the randomization groups in a constrained linear mixed model with unstructured covariance pattern accounting for repeated measurements. The changes in the number of antihypertensive drugs (defined as beta-blockers, calcium channel blockers, renin-angiotensin inhibitors, low ceiling diuretics and mineralocorticoid-receptor antagonists) during follow-up was further compared between the randomization groups using χ^2^ test.

A 2-sided *P* of ≤0.05 was considered statistically significant. Data management and statistical analyses were performed using R (version 4.1.0).

## Results

Of 6004 LOOP participants, 5997 were included in the present study, with an average blood pressure of 150 mm Hg. Figure S1 presents the distribution of baseline SBP. Baseline characteristics for participants with SBP ≥150 mm Hg and <150 mm Hg are summarized in Table 1. Participants with SBP ≥150 mm Hg were significantly older and had lower heart rates, less tobacco exposure, higher alcohol consumption and marginally lower CHA_2_DS_2_-VASc score. Although a medical history of hypertension was more prevalent in participants with SBP≥150 mm Hg, there was no significant difference in the number of antihypertensive drugs compared with those having lower SBP. Among participants with SBP ≥150 mm Hg, only 92.8% had a prior diagnosis of hypertension at baseline, while 11.3% received no antihypertensive treatment.

**Table 1.**
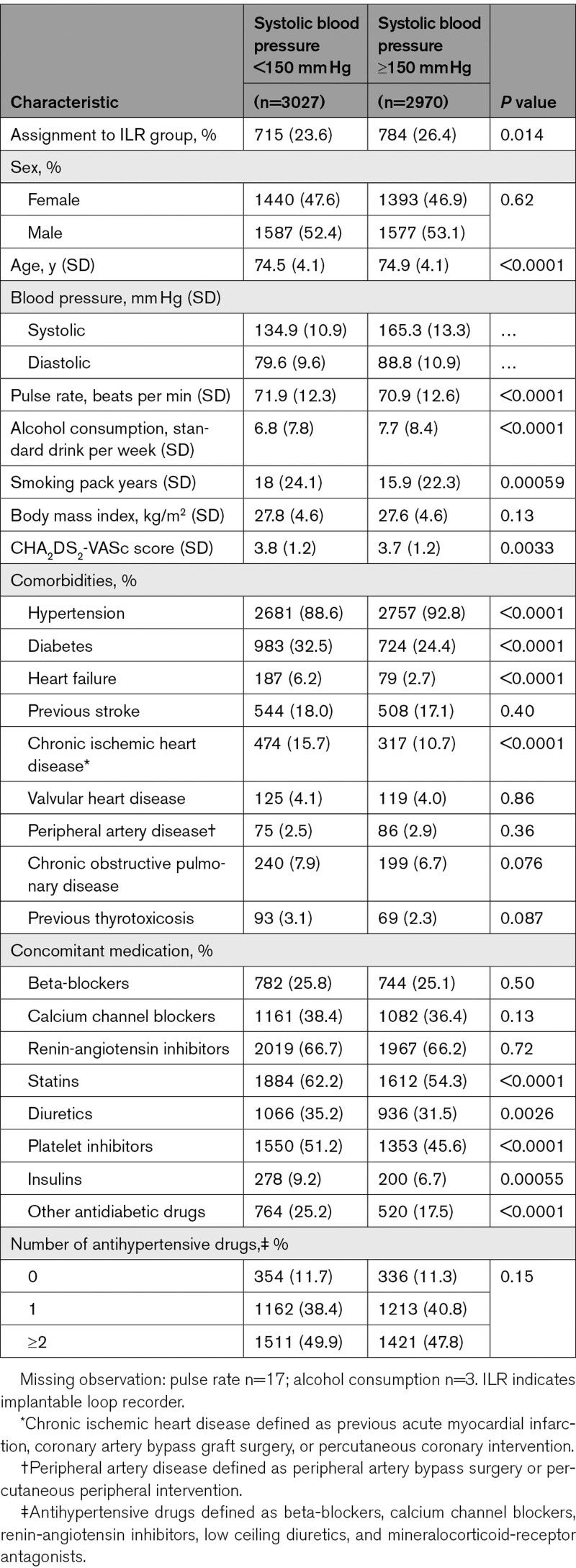
Overview of Baseline Characteristics Stratified by Systolic Blood Pressure ≥150 mm Hg

**Figure 1.**
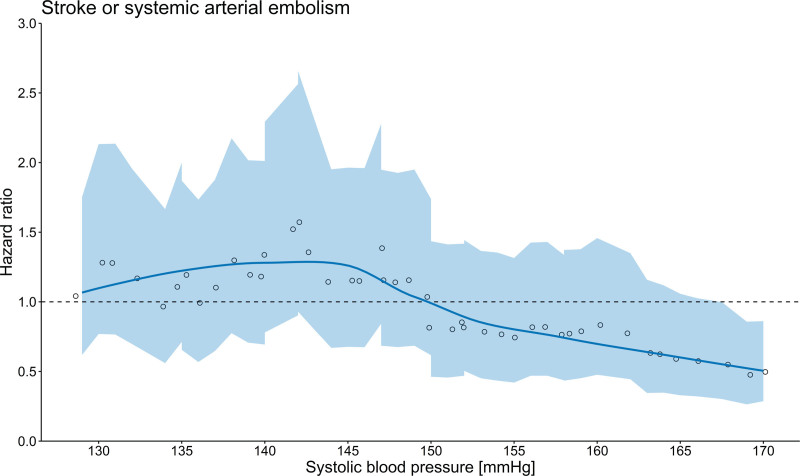
**Relation between systolic blood pressure and implantable loop recorder (ILR) screening efficacy on the primary outcome.** The *y*-axis shows the hazard ratios of stroke or systemic arterial embolism in the ILR group compared with the control group. Hazard ratio was estimated for each of sequentially overlapping subpopulations created by a sliding window approach iterating through increasing systolic blood pressure and plotted as circle, with the median systolic blood pressure as the x-axis coordinate. The colored area represents the exact 95% CIs. Based on the estimated hazard ratios and corresponding median blood pressures, a polynomial, moving-average regression plot with locally estimated smoothing was constructed.

Figure 1 shows the relation between SBP at enrolment and the effect of ILR screening on the primary outcome. The HR of stroke or systemic arterial embolism for the ILR group versus the control group decreased with increasing SBP. At a value of 150 mm Hg, the trend curve crossed the reference line that represented an equal risk of the events in the ILR group as the control group. The threshold of SBP≥150 mm Hg was therefore selected for subsequent outcome analyses.

### The Primary and Secondary Outcomes

**Figure 2.**
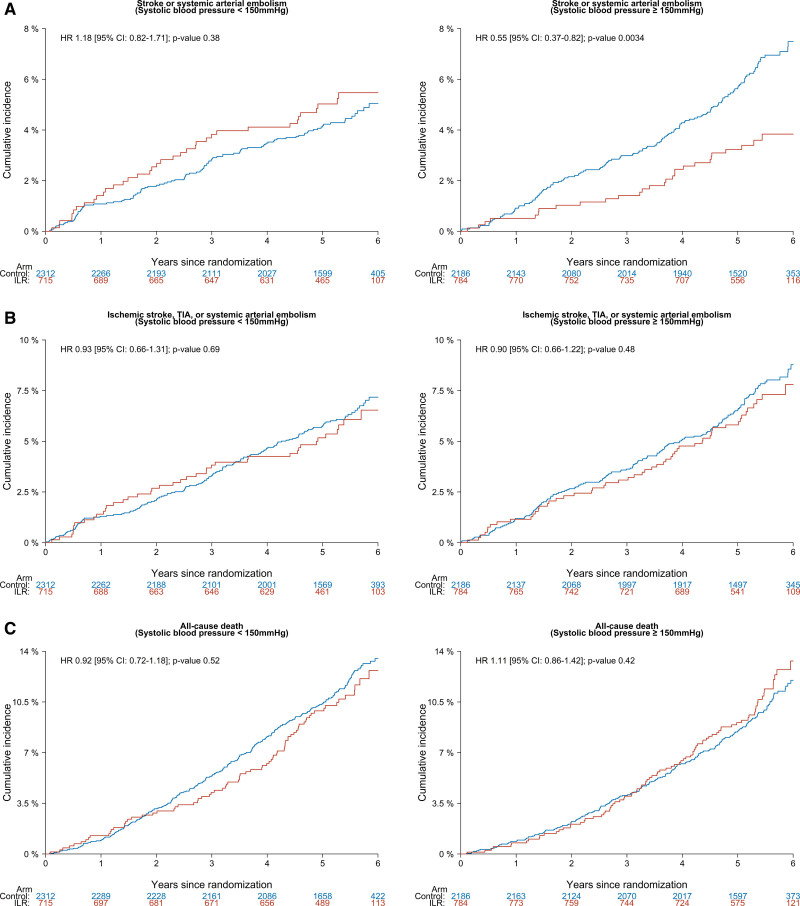
**Cumulative incidence of primary and secondary outcomes in participants with systolic blood pressure <150 mmHg and ≥150 mmHg according to randomization assignment.** Cumulative incidence cures for stroke of systemic arterial embolism (**A**), ischemic stroke, TIA, or systemic arterial embolism (**B**), and all-cause death (**C**) according to randomization assignment, stratified by baseline systolic blood pressure <150 mmHg (Left) or ≥150 mmHg (Right). HR indicates hazard ratio; ILR, implantable loop recorder; and TIA, transient ischemic attack.

Among participants with SBP<150 mm Hg, the ILR group did not differ from the control group significantly with respect to the risks of the primary or secondary outcomes (Figure 2; Table 2). For participants with SBP≥150 mm Hg, a remarkably lower event rate of stroke or systemic arterial embolism was reported in the ILR group (0.72 events per 100 person-years [95% CI, 0.48–1.03]) than the control group (1.30 events per 100 person-years [95% CI, 1.10–1.53]); HR, 0.55 [95% CI, 0.37–0.82]. This remained true after multivariate adjustment (adjusted HR, 0.56 [95% CI, 0.37–0.83]). For the composite of ischemic stroke, transient ischemic attack, or systemic arterial embolism, these events were also less likely to occur in the ILR group (1.36 events per 100 person-years [95% CI, 1.02–1.77]) compared with the control group (1.52 events per 100 person-years [95% CI, 1.30–1.76]), but the difference was not significant (adjusted HR, 0.92 [95% CI, 0.68–1.25]). For all-cause death, no significant benefit was achieved by ILR screening compare with usual care (adjusted HR, 1.13 [95% CI, 0.88–1.45]).

**Table 2.**
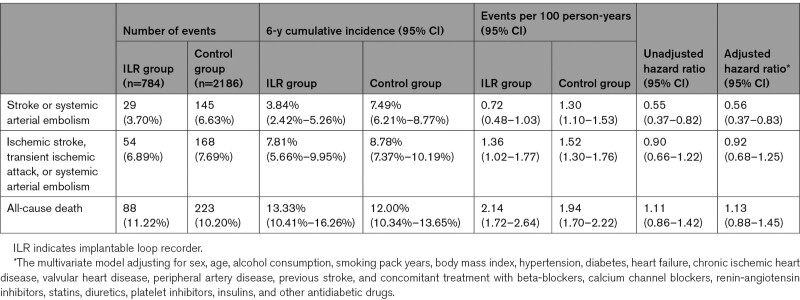
Primary and Secondary Outcomes in Participants With Systolic Blood Pressure ≥150 mm Hg

Cumulative incidences of hemorrhagic stroke and ischemic thromboembolism within the primary outcome stratified by SBP are depicted in Figure S2. Among participants with SBP<150 mm Hg, the hemorrhagic and the ischemic events occurred at comparable incidence rates in both randomization groups (*P* 0.15 and 0.69, respectively). Likewise, the occurrence of hemorrhagic stroke did not differ noticeably between the randomization groups among participants with higher blood pressure either (*P*, 0.32). In contrast, a significant reduction of the ischemic events within the primary outcome was observed in participants with SBP≥150 mm Hg by ILR screening (*P*, 0.0046). Similar results were yielded by multivariate modelling (Table S1).

### AF Subtype and Duration

**Figure 3.**
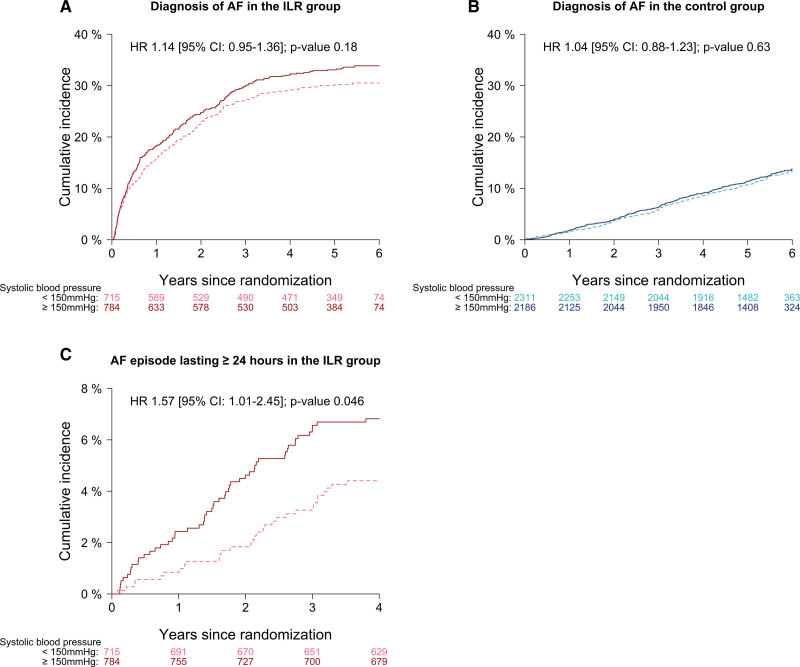
**Cumulative incidence of atrial fibrillation (AF) diagnosis and AF episodes lasting ≥24 hours according to systolic blood pressure.** Cumulative incidence curves for AF diagnosis in each randomization group (**A** and **B**), and for AF episodes ≥24 hours in the implantable loop recorder (ILR) group (**C**) according to baseline systolic blood pressure <150 mmHg (dashed lines) or ≥150 mmHg (solid lines). HR indicates hazard ratio.

In the control group, the event rates of AF diagnosis were indifferent across the SBP risk strata (HR, 1.04 [95% CI, 0.88–1.23]; Figure 3). For the ILR group, albeit a relatively greater increase in the incidence of AF diagnosis among participants with SBP≥150 mm Hg (8.55 events per 100 person-years [95% CI, 7.55–9.65]) compared with those having lower blood pressure (7.52 events per 100 person-years [95% CI, 6.54–8.59]), no statistical significance was reached (HR, 1.14 [95% CI, 0.95–1.36]). These results were robust in the multivariate analysis. In terms of longer AF episodes, SBP≥150 mm Hg was associated with significantly increased risk of AF episodes ≥24 hours detected by ILR (HR, 1.57 [95% CI, 1.01–2.45]; adjusted HR, 1.70 [95% CI, 1.08–2.69]).

Further exploration of the interaction between SBP and AF diagnosis are illustrated in Figures S3 and S4. In the control group, the event risk was increasing with elevating SBP from the reference of 130 mm Hg (*P*, 0.010; adjusted *P*, 0.029). When SBP was assessed in relation to AF diagnosis in the ILR group, this positive correlation appeared to be diminished and became nonsignificant (*P*, 0.73; adjusted *P*, 0.69).

### Blood Pressure Management

**Figure 4.**
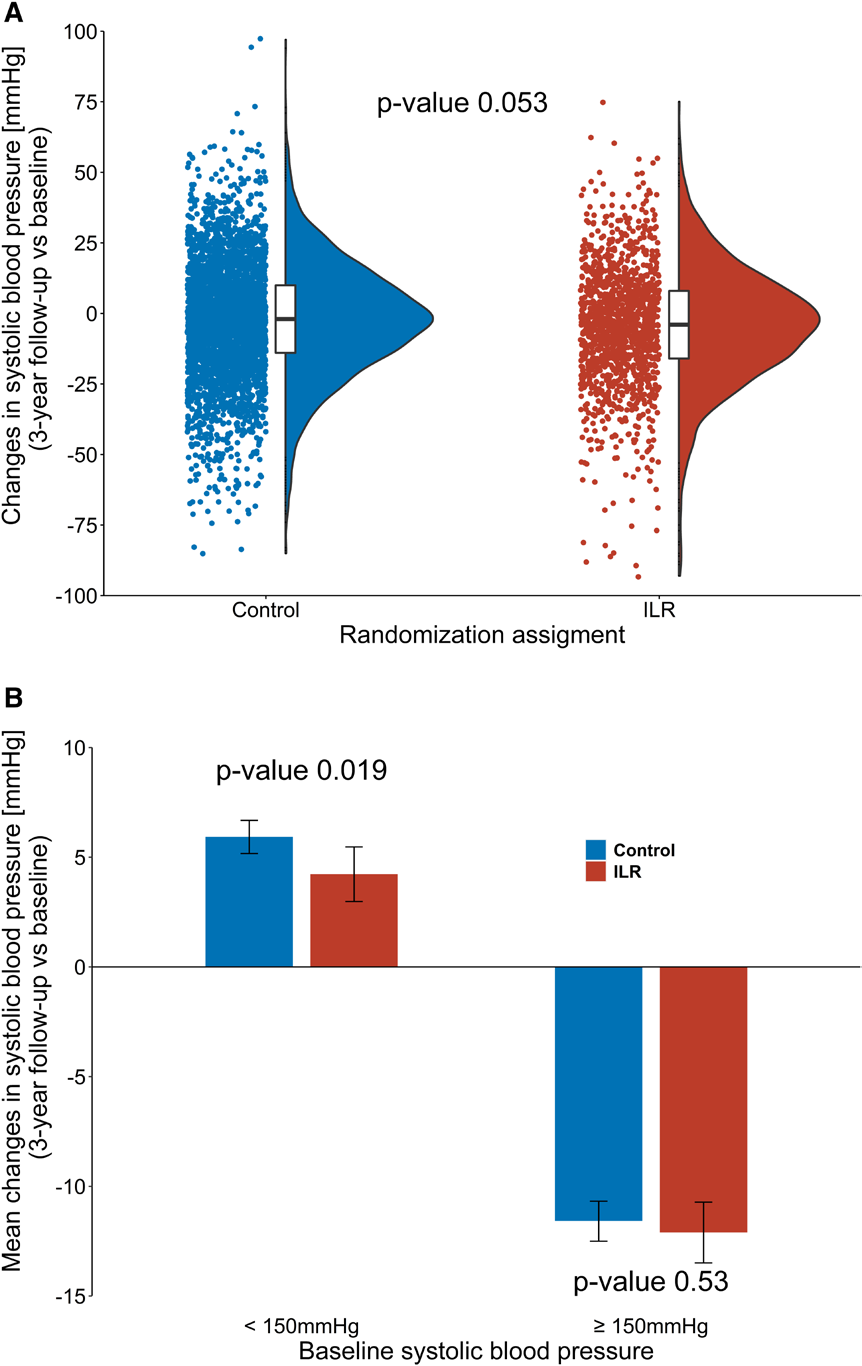
**Changes in systolic blood pressure at 3-year follow-up.** The figure shows the distribution of 3-year changes in systolic blood pressure according to randomization assignment (**A**) and the mean changes in systolic blood pressure according to randomization assignment in participants with <150 and ≥150 mm Hg at baseline (**B**). Error bars present 95% CIs of mean blood pressure changes. ILR indicates implantable loop recorder.

Of 5997 participants included, 4946 (82.5%) had available blood pressure measurements at 3-year follow-up. The mean SBP reduction for 3-year follow-up versus baseline was 2.74 mm Hg [95% CI, 2.11–3.37] in the control group and 3.80 mm Hg [95% CI, 2.84–4.76] in the ILR group (*P*, 0.053; Figure 4). Among participants with baseline SBP <150 mm Hg, a mean blood pressure increase of 5.93 mm Hg [95% CI, 5.17–6.68] was observed at 3-year follow-up in the control group and 4.23 mm Hg [95% CI, 2.98–5.47] in the ILR group; *P*, 0.019. For participants having SBP ≥150 mm Hg at baseline, no significant changes in blood pressure was found between the control group (mean SBP reduction of 11.58 mm Hg [95% CI, 10.68–12.50]) and the ILR group (mean SBP reduction of 12.10 mm Hg [95% CI, 10.72–13.49]); *P*, 0.53. Additionally, for changes in the number of antihypertensive drugs during follow-up, there was no remarkable difference between the randomization groups among participants with SBP ≥150 mm Hg (*P*, 0.22; Table S2).

## Discussion

This post hoc analysis of the LOOP Study investigated the interaction between SBP and the effects of continuous AF screening in elderly, at-risk individuals. The principal findings were as follows: (1) the ILR screening benefit on the risk of stroke or systemic arterial embolism increased with elevating SBP, where an almost 50% risk reduction was obtained by screening participants with SBP ≥150 mm Hg; (2) higher SBP was associated with increasing incidence of AF diagnosis in the control group, but did not affect the overall AF occurrence detected by ILR; and (3) SBP ≥150 mm Hg was associated with >1.5-fold increased risk of ILR-detected AF episodes ≥24 hours compared with lower blood pressure.

To date, only 2 randomized trials have evaluated the effects of AF screening on clinical end points. In the study STROKESTOP (Systematic ECG Screening for AF Among 75 Year Old Subjects in the Region of Stockholm and Halland, Sweden) with intermittent single-lead ECG screening of individuals aged 75 to 76 years twice daily for 2 weeks, a marginal reduction of the composite end point of stroke, systemic embolism, hospitalization for bleeding, and all-cause mortality was reported,^19^ whereas the main analysis of the LOOP Study showed a nonsignificant 20% decrease in stroke risk by ILR screening.^7^ These results highlight the need of more refined risk stratification schemes with regard to screening for subclinical AF and subsequent anticoagulation. In the present analysis of the LOOP Study, SBP demonstrated promising results in predicting stroke prevention with ILR screening, as the screening benefits increased with increasing blood pressure. This suggests that blood pressure may influence the stroke risk of subclinical AF in similar way as that of clinical AF. Among participants with SBP ≥150 mm Hg, ILR screening was associated with a significant reduction in stroke and systemic arterial embolism. This was primarily driven by a considerable decrease in ischemic stroke and systemic arterial embolism, which may be attributable to oral anticoagulation initiation upon AF detection. Indeed, it seems unlikely that the reduced risk of stroke in the ILR group was caused by potentially better blood pressure management, as no significant difference in SBP changes for 3-year follow-up versus baseline was detected between the randomization groups among participants with SBP ≥150 mm Hg. Neither did the changes in the number of antihypertensive drugs during follow-up differ significantly between the randomization groups among these participants.

Several studies have pointed to an association between elevated SBP and increased AF risk.^10,14,15^ In the Atherosclerosis Risk in Communities (ARIC) cohort, Huxley et al^10^ reported SBP ≥140 mm Hg as being the contributor to the majority of incident AF cases, while a lower blood pressure resulted in 35% to 45% risk reduction. A post hoc analysis of the Framingham Heart Study concluded that hypertensive individuals were predisposed to double the risk of AF as in those normotensive.^15^ Consistently with these studies, we also found a positive correlation between SBP and AF diagnosis in the control group that may represent a valid estimate of incident, clinical AF in the background population. Nevertheless, no significant association was detected between SBP and AF diagnosis in the ILR group. Given the excellent ability of ILR monitoring to detect AF (except the very brief ones), our result might imply that SBP has no impact on the overall AF development. This finding is contrary to the study Prevalence of Sub-Clinical AF Using an Implantable Cardiac Monitor (ASSERT-II), wherein Healey et al^16^ observed 11% lowered risk of device-detected AF≥5 minutes with every 10 mm Hg increment of SBP. The discrepancy might be explained by more comorbidity in the ASSERT-II population due to their inclusion criteria and recruitment from cardiological and neurological clinics opposed to outside the clinical setting in the LOOP Study. Additionally, more specialized care could have led to a higher probability of early, incidentally diagnosed AF especially in individuals with high blood pressure, which would exclude them from enrolment in ASSERT-II. Notwithstanding the lacking relation between SBP and the overall development of AF, we observed a >1.5-fold increased risk of ILR-detected AF episodes ≥24 hours in participants with SBP ≥150 mm Hg, compared with those having lower blood pressure. Longer AF duration has been linked to increased thromboembolic risk by numerous prior studies.^18,20–23^ In a study of AF patients with pacemaker, Capucci et al^23^ demonstrated that device-detected AF recurrences >24 hours conferred an increased risk of embolic events compared with shorter AF episodes or no recurrence. Furthermore, a post hoc analysis of the ASSERT reported that only device-detected AF episodes >24 hours were associated with increased thromboembolic risk in patients with cardiovascular implantable electronic devices, but not those between 6 minutes and 24 hours.^18^ Hence, the observed relation between higher SBP and longer AF episodes might partly explain why ILR screening for subclinical AF was less beneficial among participants with lower blood pressure. This also corroborates the link between high SBP and increased stroke risk in individuals with known AF as previously ascertained.^11–13^

### Limitations

Several limitations exist in the present study. First, the exploratory nature of the analysis limited our results to be solely hypothesis-generating. Second, data on other stroke risk factor such as body mass index and physical activity during follow-up are lacking and more frequent study visit in the ILR group could have led to better management of these, especially among individuals with high blood pressure. Third, baseline SBP was a single-timepoint reading and might not represent a valid picture of the participants’ daily blood pressure levels. Fourth, acknowledgement of high blood pressure at enrolment could have led to more visits to general practitioners and thereby increased incidental diagnosis of AF in the control group. Fifth, we only have data on prescribed drugs, but not on drug compliance. Sixth, only 82.5% of the study participants had available SBP measurement at 3-year follow-up and hence, a potentially significant difference in SBP changes between the randomization groups might be undiscovered. Seventh, data on cardiac morphological parameters such as left atrial enlargement or ventricular hypertrophy are lacking.

### Perspectives

Our study suggests that AF screening in elderly, at-risk individuals with SBP≥150 mm Hg could lead to substantial stroke risk reduction. However, as blood pressure is a modifiable risk factor, a dichotomous screening threshold would likely be too arbitrary in clinical practice. Rather, physicians should pay more attention on minimizing health risks of subclinical AF by better management of blood pressure. Moreover, as the majority of our study population had a baseline history of hypertension, our findings would emphasize the need for more research in AF screening with respect to dysregulated hypertension.

### Conclusions

In an elderly, at-risk population, ILR screening for AF was associated with a significant reduction in stroke or systemic arterial embolism among participants with SBP ≥150 mm Hg and the screening benefit increased with increasing blood pressure. High SBP did not affect the overall AF occurrence, but it was associated with increased risk of longer AF episodes which might explain the positive correlation between blood pressure and the screening effects. However, these findings should be considered as hypothesis-generating and warrant further study.

## Article Information

### Acknowledgments

We thank Christian Kronborg (University of Southern Denmark, Denmark) for his contribution in the Trial Steering Committee of the LOOP Study. We thank Dan Atar (University of Oslo and Department of Cardiology B, Oslo University Hospital Ullevål, Norway), Gregory Y. H. Lip (The University of Liverpool and Liverpool Heart and Chest Hospital, United Kingdom), and Mårten Rosenqvist (Karolinska Institutet and Danderyd Hospital, Sweden), for assisting the LOOP study with their expertise in the international advisory committee. We thank the research nurses and other colleagues in the Departments of Cardiology at Rigshospitalet, Bispebjerg and Frederiksberg Hospital, Zealand University Hospital, and Odense University Hospital who assisted with the conduct of the LOOP Study.

### Sources of Funding

The LOOP Study was supported by Innovation Fund Denmark (grant number 12-1352259), The Research Foundation for the Capital Region of Denmark, The Danish Heart Foundation (grant number 11-04-R83-A3363-22625), Aalborg University Talent Management Program, Arvid Nilssons Fond, Skibsreder Per Henriksen, R og Hustrus Fond, the European Union’s Horizon 2020 program (grant number 847770 to the AFFECT-EU consortium), Læge Sophus Carl Emil Friis og hustru Olga Doris Friis’ Legat, and an unrestricted grant from Medtronic. The employment of the first author, L.Y. Xing, is funded by the AFFECT-EU consortium and thereby the European Union’s Horizon 2020 program (grant number 847770).

### Disclosures

J.H. Svendsen reports to be a member of Medtronic advisory boards and to have received speaker honoraria and research grants from Medtronic in relation to this work and outside this work. S.Z. Diederichsen reports to be a part-time employee of Vital Beats and advisor at Bristol-Myers Squibb/Pfizer, not related to this work. D.W. Krieger reports to be a Medtronic Focus Group member. A. Brandes reports research grants from The Region of Southern Denmark and The Region of Zealand, The Canadian Institutes of Health Research, and Theravance, and speaker honoraria from Bayer, Boehringer Ingelheim, and Bristol-Myers Squibb, and a travel grant from Biotronik not related to this work. K.J. Haugan reports travel and educational grants from Medtronic, Abbott, and Biotronik and speaker honoraria from Boehringer-Ingelheim not related to this work. L. Køber reports speaker honoraria from Novo, AstraZeneca, Novartis, and Boehringer, not related to this work. L.Y. Xing, S. Højberg, C. Graff, and M.S. Olesen report no conflicts.

### Supplemental Material

Tables S1 and S2

Figures S1–S4

## Supplementary Material



## References

[1] Lip GY Lane DA. Stroke prevention in atrial fibrillation: a systematic review. JAMA. 2015;313:1950–1962. doi: 10.1001/jama.2015.4369 25988464 10.1001/jama.2015.4369

[2] Wolf PA Abbott RD Kannel WB. Atrial fibrillation as an independent risk factor for stroke: the framingham study. Stroke. 1991;22:983–988. doi: 10.1161/01.str.22.8.983 1866765 10.1161/01.str.22.8.983

[3] Hindricks G Potpara T Dagres N Arbelo E Bax JJ Blomström-Lundqvist C Boriani G Castella M Dan GA Dilaveris PE; ESC Scientific Document Group. 2020 ESC Guidelines for the diagnosis and management of atrial fibrillation developed in collaboration with the European Association for Cardio-Thoracic Surgery (EACTS): The Task Force for the diagnosis and management of atrial fibrillation of the European Society of Cardiology (ESC) Developed with the special contribution of the European Heart Rhythm Association (EHRA) of the ESC. Eur Heart J. 2021;42:373–498. doi: 10.1093/eurheartj/ehaa612 32860505 10.1093/eurheartj/ehaa612

[4] Hart RG Pearce LA Aguilar MI. Meta-analysis: antithrombotic therapy to prevent stroke in patients who have nonvalvular atrial fibrillation. Ann Intern Med. 2007;146:857–867. doi: 10.7326/0003-4819-146-12-200706190-00007 17577005 10.7326/0003-4819-146-12-200706190-00007

[5] January CT Wann LS Calkins H Chen LY Cigarroa JE Cleveland JC Jr Ellinor PT Ezekowitz MD Field ME Furie KL. 2019 AHA/ACC/HRS focused update of the 2014 AHA/ACC/HRS guideline for the management of patients with atrial fibrillation: a report of the American College of Cardiology/American Heart Association Task Force on Clinical Practice Guidelines and the Heart Rhythm Society in Collaboration with the Society of Thoracic Surgeons. Circulation. 2019;140:e125–e151. doi: 10.1161/CIR.0000000000000665 30686041 10.1161/CIR.0000000000000665

[6] Healey JS Connolly SJ Gold MR Israel CW Van Gelder IC Capucci A Lau CP Fain E Yang S Bailleul C; ASSERT Investigators. Subclinical atrial fibrillation and the risk of stroke. N Engl J Med. 2012;366:120–129. doi: 10.1056/NEJMoa1105575 22236222 10.1056/NEJMoa1105575

[7] Svendsen JH Diederichsen SZ Højberg S Krieger DW Graff C Kronborg C Olesen MS Nielsen JB Holst AG Brandes A. Implantable loop recorder detection of atrial fibrillation to prevent stroke (The LOOP Study): a randomised controlled trial. Lancet. 2021;398:1507–1516. doi: 10.1016/S0140-6736(21)01698-6 34469766 10.1016/S0140-6736(21)01698-6

[8] Rapsomaniki E Timmis A George J Pujades-Rodriguez M Shah AD Denaxas S White IR Caulfield MJ Deanfield JE Smeeth L. Blood pressure and incidence of twelve cardiovascular diseases: lifetime risks, healthy life-years lost, and age-specific associations in 1·25 million people. Lancet. 2014;383:1899–1911. doi: 10.1016/S0140-6736(14)60685-1 24881994 10.1016/S0140-6736(14)60685-1 PMC4042017

[9] Benjamin EJ Levy D Vaziri SM D’Agostino RB Belanger AJ Wolf PA. Independent risk factors for atrial fibrillation in a population-based cohort. The Framingham Heart Study. JAMA. 1994;271:840–844. 8114238

[10] Huxley RR Lopez FL Folsom AR Agarwal SK Loehr LR Soliman EZ Maclehose R Konety S Alonso A. Absolute and attributable risks of atrial fibrillation in relation to optimal and borderline risk factors: the Atherosclerosis Risk in Communities (ARIC) study. Circulation. 2011;123:1501–1508. doi: 10.1161/CIRCULATIONAHA.110.009035 21444879 10.1161/CIRCULATIONAHA.110.009035 PMC3181498

[11] Van Staa TP Setakis E Di Tanna GL Lane DA Lip GYH. A comparison of risk stratification schemes for stroke in 79 884 atrial fibrillation patients in general practice. J Thromb Haemost. 2011;9:39–48. doi: 10.1111/j.1538-7836.2010.04085.x 21029359 10.1111/j.1538-7836.2010.04085.x

[12] Hart RG Pearce LA McBride R Rothbart RM Asinger RW. Factors associated with ischemic stroke during aspirin therapy in atrial fibrillation: analysis of 2012 participants in the SPAF I-III clinical trials. The Stroke Prevention in Atrial Fibrillation (SPAF) Investigators. Stroke. 1999;30:1223–1229. doi: 10.1161/01.str.30.6.1223 10356104 10.1161/01.str.30.6.1223

[13] Böhm M Brueckmann M Eikelboom JW Ezekowitz M Fräßdorf M Hijazi Z Hohnloser SH Mahfoud F Schmieder RE Schumacher H. Cardiovascular outcomes, bleeding risk, and achieved blood pressure in patients on long-term anticoagulation with the thrombin antagonist dabigatran or warfarin: data from the RE-LY trial. Eur Heart J. 2020;41:2848–2859. doi: 10.1093/eurheartj/ehaa247 32385506 10.1093/eurheartj/ehaa247

[14] Sharashova E Wilsgaard T Ball J Morseth B Gerdts E Hopstock LA Mathiesen EB Schirmer H Løchen ML. Long-term blood pressure trajectories and incident atrial fibrillation in women and men: the Tromsø Study. Eur Heart J. 2020;41:1554–1562. doi: 10.1093/eurheartj/ehz234 31050731 10.1093/eurheartj/ehz234 PMC7174044

[15] Rahman F Yin X Larson MG Ellinor PT Lubitz SA Vasan RS McManus DD Magnani JW Benjamin EJ. Trajectories of risk factors and risk of new-onset atrial fibrillation in the framingham heart study. Hypertension. 2016;68:597–605. doi: 10.1161/HYPERTENSIONAHA.116.07683 27512109 10.1161/HYPERTENSIONAHA.116.07683 PMC4982514

[16] Healey JS Alings M Ha A Leong-Sit P Birnie DH de Graaf JJ Freericks M Verma A Wang J Leong D; ASSERT-II Investigators. Subclinical atrial fibrillation in older patients. Circulation. 2017;136:1276–1283. doi: 10.1161/CIRCULATIONAHA.117.028845 28778946 10.1161/CIRCULATIONAHA.117.028845

[17] Diederichsen SZ Haugan KJ Køber L Højberg S Brandes A Kronborg C Graff C Holst AG Nielsen JB Krieger D. Atrial fibrillation detected by continuous electrocardiographic monitoring using implantable loop recorder to prevent stroke in individuals at risk (the LOOP study): Rationale and design of a large randomized controlled trial. Am Heart J. 2017;187:122–132. doi: 10.1016/j.ahj.2017.02.017 28454796 10.1016/j.ahj.2017.02.017

[18] Van Gelder IC Healey JS Crijns HJGM Wang J Hohnloser SH Gold MR Capucci A Lau CP Morillo CA Hobbelt AH. Duration of device-detected subclinical atrial fibrillation and occurrence of stroke in ASSERT. Eur Heart J. 2017;38:1339–1344. doi: 10.1093/eurheartj/ehx042 28329139 10.1093/eurheartj/ehx042

[19] Svennberg E Friberg L Frykman V Al-Khalili F Engdahl J Rosenqvist M. Clinical outcomes in systematic screening for atrial fibrillation (STROKESTOP): a multicentre, parallel group, unmasked, randomised controlled trial. Lancet. 2021;398:1498–1506. doi: 10.1016/S0140-6736(21)01637-8 34469764 10.1016/S0140-6736(21)01637-8

[20] Botto GL Padeletti L Santini M Capucci A Gulizia M Zolezzi F Favale S Molon G Ricci R Biffi M. Presence and duration of atrial fibrillation detected by continuous monitoring: crucial implications for the risk of thromboembolic events. J Cardiovasc Electrophysiol. 2009;20:241–248. doi: 10.1111/j.1540-8167.2008.01320.x 19175849 10.1111/j.1540-8167.2008.01320.x

[21] Glotzer TV Daoud EG Wyse DG Singer DE Ezekowitz MD Hilker C Miller C Qi D Ziegler PD. The Relationship between daily atrial tachyarrhythmia burden from implantable device diagnostics and stroke risk the trends study. Circ Arrhythm Electrophysiol. 2009;2:474–480. doi: 10.1161/CIRCEP.109.849638 19843914 10.1161/CIRCEP.109.849638

[22] Perino AC Fan J Askari M Heidenreich PA Keung E Raitt MH Piccini JP Ziegler PD Turakhia MP. Practice variation in anticoagulation prescription and outcomes after device-detected atrial fibrillation: insights from the veterans health administration. Circulation. 2019;139:2502–2512. doi: 10.1161/CIRCULATIONAHA.118.038988 30880434 10.1161/CIRCULATIONAHA.118.038988 PMC6652191

[23] Capucci A Santini M Padeletti L Gulizia M Botto G Boriani G Ricci R Favale S Zolezzi F Di Belardino N; Italian AT500 Registry Investigators. Monitored atrial fibrillation duration predicts arterial embolic events in patients suffering from bradycardia and atrial fibrillation implanted with antitachycardia pacemakers. J Am Coll Cardiol. 2005;46:1913–1920. doi: 10.1016/j.jacc.2005.07.044 16286180 10.1016/j.jacc.2005.07.044

