# Supplementary material for: Systolic Blood Pressure and Effects of Screening for Atrial Fibrillation With Long-Term Continuous Monitoring (a LOOP Substudy)
Source: Hypertension. 2022 Jul 8;79(9):2081–90. doi: 10.1161/HYPERTENSIONAHA.122.19333 (PMC9370254; doi:10.1161/HYPERTENSIONAHA.122.19333)
Supplement: Supplementary file 1 [file hyp-79-2081-s001.pdf]

SUPPLEMENTAL MATERIAL

Systolic Blood Pressure and Effects of Screening for Atrial Fibrillation with Long-term Continuous Monitoring (a LOOP Sub-Study)

Lucas Yixi XING, MD[a]; Søren Zöga DIEDERICHSEN, MD, PhD[a,b]; Søren HØJBERG, MD, PhD[b]; Derk W. KRIEGER, MD, PhD[c,d]; Claus GRAFF, MSc, PhD[e]; Morten S. OLESEN, MSc, PhD[a,f]; Axel BRANDES, MD, DMSc[g,h]; Lars KØBER, MD, DMSc[a,i]; Ketil Jørgen HAUGAN, MD, PhD[j]; Jesper Hastrup SVENDSEN, MD, DMSc[a,i].

[a] Department of Cardiology, Copenhagen University Hospital – Rigshospitalet
Address: Inge Lehmanns Vej 7, 2100 Copenhagen, DENMARK
[b] Department of Cardiology, Bispebjerg Hospital, Copenhagen University Hospital
Address: Bispebjerg Bakke 23, 2400 Copenhagen DENMARK
[c] Department of Neurology, Mediclinic City Hospital
Address: Building 37 - 26th St, Dubai, United Arabic Emirates
[d] Department of Neuroscience, Mohammed Bin Rashid University of Medicine and Health Science
Address: Al Razi St, Dubai, United Arabic Emirates
[e] Department of Health Science and Technology, Aalborg University
Address: Fredrik Bajers Vej 7 D2, 9220 Aalborg, DENMARK
[f] Department of Biomedical Sciences, Faculty of Health and Medical Sciences, University of Copenhagen
Address: Blegdamsvej 3B, 2200 Copenhagen, DENMARK
[g] Department of Clinical Research, Faculty of Health Sciences, University of Southern Denmark
Address: Winsløwparken 19, 5000 Odense C, DENMARK
[h] Department of Cardiology, Odense University Hospital
Address: J. B. Winsløws Vej 4, 5000 Odense, DENMARK
[i] Department of Clinical Medicine, Faculty of Health and Medical Sciences, University of Copenhagen
Address: Blegdamsvej 3B, 2200 Copenhagen, DENMARK
[j] Department of Cardiology, Zealand University Hospital Roskilde
Address: Sygehusvej 10, 4000 Roskilde DENMARK

Corresponding Author:
Jesper Hastrup Svendsen, MD, DMSc
Department of Cardiology, Copenhagen University Hospital – Rigshospitalet
Inge Lehmanns Vej 7, 2100 Copenhagen, DENMARK
Mail: Jesper.Hastrup.Svendsen@regionh.dk
Cell: +45 3545 8061

Table S1: The hemorrhagic and the ischemic events within the primary outcome according to randomization assignments in participants with systolic blood pressure ≥150 mmHg and <150 mmHg

		Cumulative incidence rate at 6 years (95% CI)		Events per 100 person-years (95% CI)		Adjusted hazard ratio (95% CI)*
		ILR group	Control group	ILR group	Control group	
≥150 mmHg	Ischemic stroke or systemic arterial embolism	3.21% (1.94-4.48%)	6.66% (5.43-7.88%)	0.62 (0.40-0.91)	1.15 (0.96-1.36)	0.54 (0.35-0.84)
	Hemorrhagic stroke	0.63% (0.33-1.27%)	0.83% (0.43-1.23%)	0.10 (0.03-0.25)	0.15 (0.09-0.24)	0.62 (0.21-1.87)
<150 mmHg	Ischemic stroke or systemic arterial embolism	4.48% (2.90-6.06%)	4.51% (3.55-5.47%)	0.87 (0.59-1.23)	0.80 (0.65-0.98)	1.05 (0.70-1.59)
	Hemorrhagic stroke	0.99% (0.26-1.72%)	0.53% (0.21-0.86%)	0.20 (0.08-0.40)	0.10 (0.05-0.18)	1.81 (0.70-4.67)

*The multivariate model adjusting for sex, age, alcohol consumption, smoking pack years, body mass index, hypertension, diabetes mellitus, heart failure, chronic ischemic heart disease, valvular heart disease, peripheral artery disease, previous stroke, and concomitant treatment with beta-blockers, calcium channel blockers, renin-angiotensin inhibitors, statins, diuretics, platelet inhibitors, insulins, and other antidiabetic drugs.

Hazard ratio estimated by use of cause-specific Cox proportional hazards regression censoring for death and other subtype of the primary outcome.

Event rate estimated by Poisson regression and expressed as number of events per 100 person-years.

Abbreviation: ILR, implantable loop recorder; CI, confidence interval.

Table S2**:** Changes in the number of antihypertensive drugs during the entire study period according to randomization assignments in the overall study population, participants with systolic blood pressure ≥150 mmHg and <150 mmHg at baseline.

		**ILR**	**Control**	**p-value**
**The study population** (n=5997)				
Changes in the number of antihypertensive drugs* from baseline	≤-2	31 (2.1%)	98 (2.2%)	0.64
	-1	178 (11.9%)	525 (11.7%)	
	0	1047 (69.8%)	3088 (68.6%)	
	1	209 (13.9%)	652 (14.5%)	
	≥2	36 (2.4%)	140 (3.1%)	
**Participants with baseline systolic blood pressure ≥150 mmHg** (n=2970)				
Changes in the number of antihypertensive drugs* from baseline	≤-2	16 (2.0%)	40 (1.8%)	0.22
	-1	86 (11.0%)	227 (10.4%)	
	0	530 (67.6%)	1432 (65.5%)	
	1	132 (16.8%)	393 (18.0%)	
	≥2	20 (2.6%)	94 (4.3%)	
**Participants with baseline systolic blood pressure <150 mmHg** (n=3027)				
Changes in the number of antihypertensive drugs* from baseline	≤-2	15 (2.1%)	56 (2.4%)	0.96
	-1	92 (12.9%)	298 (12.9%)	
	0	516 (72.2%)	1654 (71.5%)	
	1	76 (10.6%)	259 (11.2%)	
	≥2	16 (2.2%)	45 (2.0%)	

*Antihypertensive drugs defined as beta-blockers, calcium channel blockers, renin-angiotensin inhibitors, low ceiling diuretics, and mineralocorticoid-receptor antagonists.
Negative values of changes indicate reduced number of antihypertensive drugs during follow-up; 0 indicates no change in medication; and positive values of change indicate increased number of antihypertensive drugs during follow-up.
Abbreviation: ILR, implantable loop recorder.

Figure S1: Distribution of systolic blood pressure in the study population from The LOOP Study (*Atrial Fibrillation detected by Continuous ECG Monitoring using Implantable Loop Recorder to prevent Stroke in High-risk Individuals*)

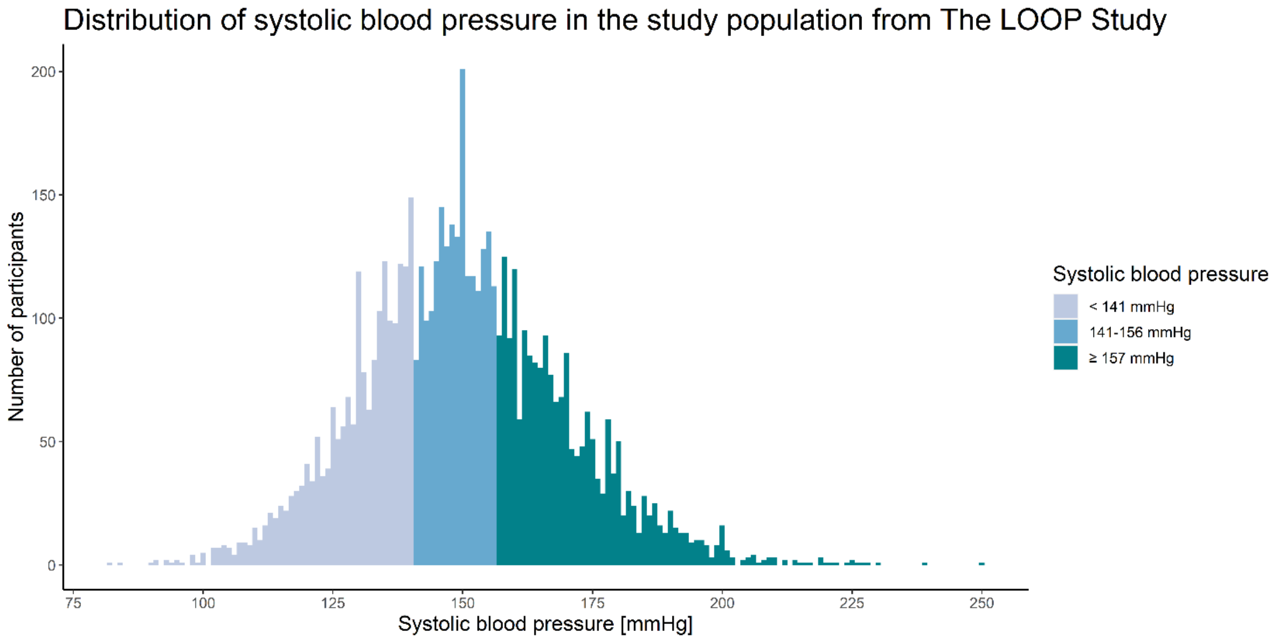

Different colors represent the tertiles of systolic blood pressure.

Figure S2: Cumulative incidence curves for types of the primary outcome in participants with systolic blood pressure <150 mmHg and ≥150 mmHg according to randomization assignment

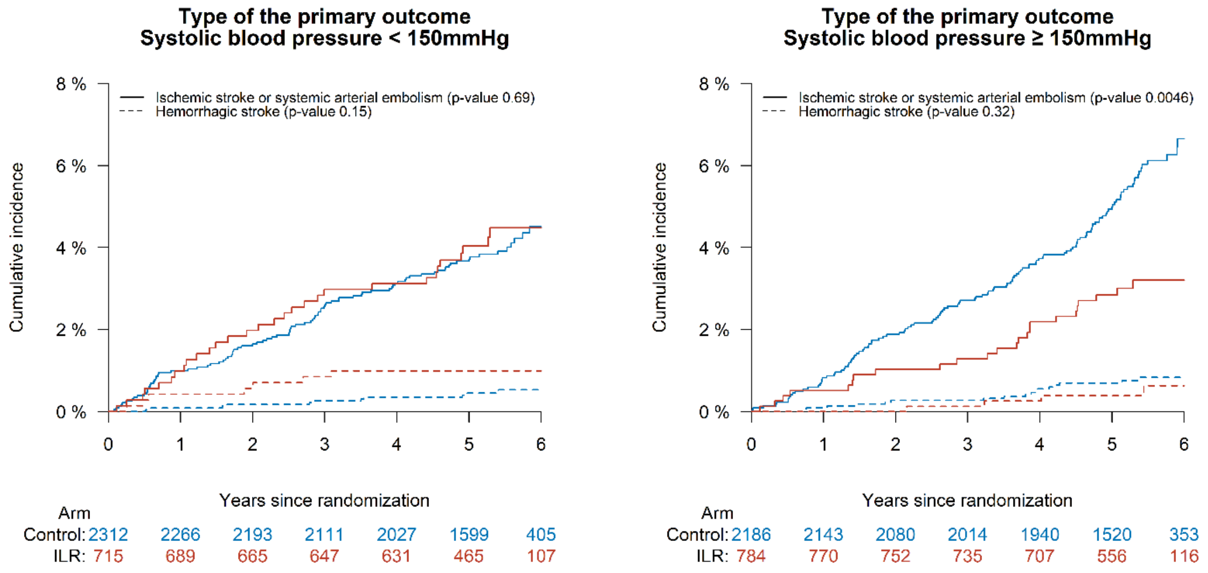

Graphical presentation of cumulative incidences for hemorrhagic stroke and ischemic thromboembolism (ischemic stroke or systemic arterial embolism) within the primary outcome stratified by systolic blood pressure ≥150 mmHg. Cumulative incidences were calculated and plotted using the Aalen-Johansen method. P-values were estimated for each subtype of the primary outcome between the randomization groups by use of cause-specific Cox proportional hazard model.

Abbreviation: ILR, implantable loop recorder.

Figure S3: AF diagnosis in the control group according to systolic blood pressure

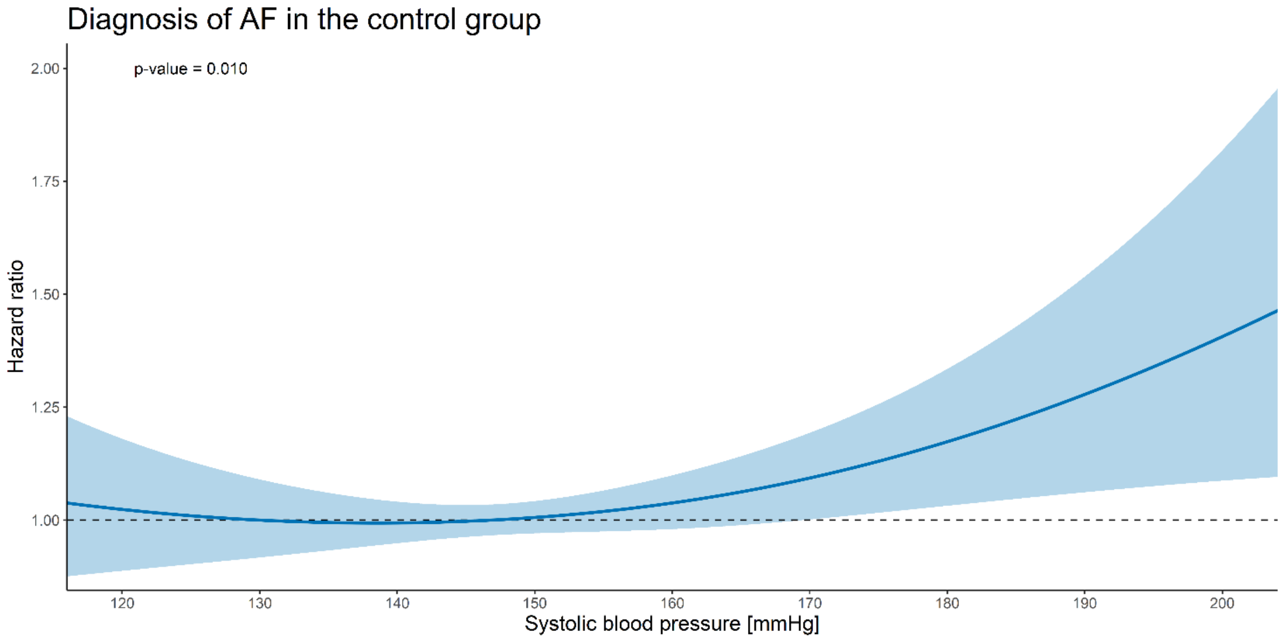

The y-axis shows the hazard ratio of AF diagnosis, while the x-axis shows systolic blood pressure in mmHg. A penalized spline model was applied to assess the relation between blood pressure and the relative risk of AF diagnosis in the control group, with hazard ratio estimated as ratio of hazard rates for each level of blood pressure compared to 130 mmHg. The colored area represents the estimated 95% confidence interval.

Abbreviation: AF, atrial fibrillation.

Figure S4: AF diagnosis in the ILR group according to systolic blood pressure

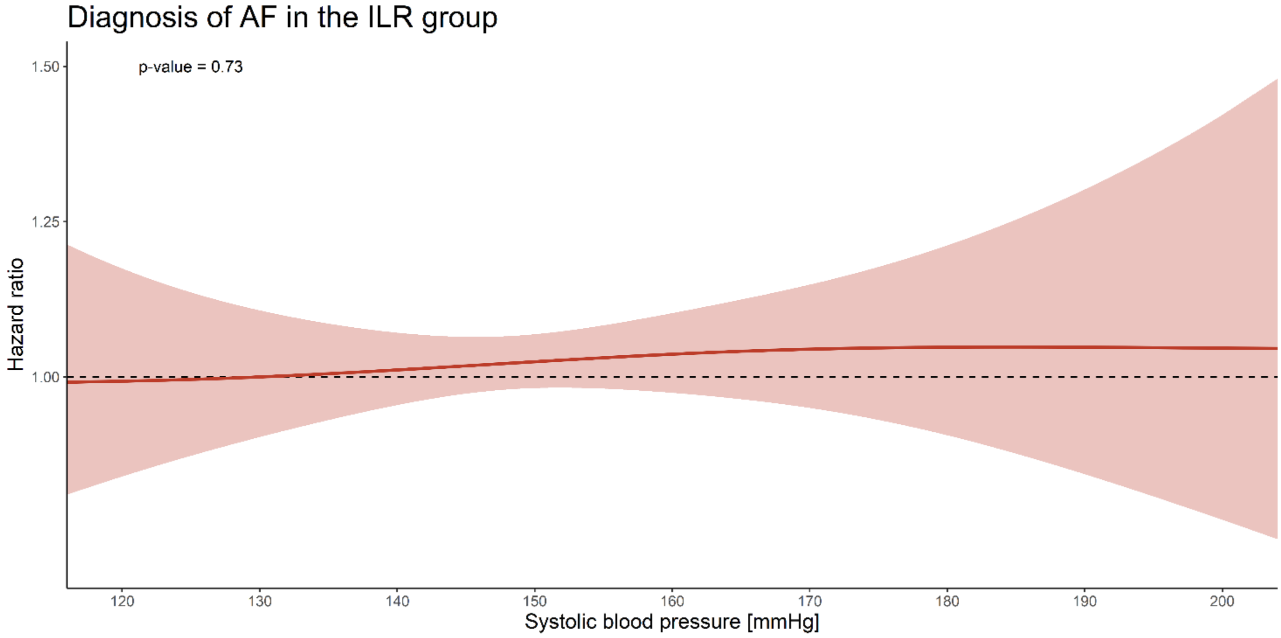

The y-axis shows the hazard ratio of AF diagnosis, while the x-axis shows systolic blood pressure in mmHg. A penalized spline model was applied to assess the relation between blood pressure and the relative risk of AF diagnosis in the ILR group, with hazard ratio estimated as ratio of hazard rates for each level of blood pressure compared to 130 mmHg. The colored area represents the estimated 95% confidence interval.

Abbreviation: AF, atrial fibrillation; ILR, implantable loop recorder.
